# Supplementary material for: Survival following sublobar resection after neoadjuvant therapy for T1N1-2M0 lung cancer
Source: PLoS One. 2026 Jun 3;21(6):e0349231. doi: 10.1371/journal.pone.0349231 (PMC13232803; doi:10.1371/journal.pone.0349231)
Supplement: S1 Table — Kaplan–Meier curves demonstrate 1‑year overall survival for patients with clinical stage II NSCLC (n = 296) treated with lobectomy or sublobar resection (overall, wedge, or anatomic segmentectomy). At baseline, 275 patients were at risk in the lobectomy cohort and 21 in the sublobar group. One‑year survival was 92.73% for lobectomy, 95.24% for the sublobar overall group, 87.5% for wedge resections, and 100% for segmentectomy. Standard errors are provided at each time interval to reflect precision of survival estimates. (DOCX) [file pone.0349231.s001.docx]

Supplemental Data — Survival Following Sublobar Resection After Neoadjuvant Therapy for T1N1–2M0 Lung Cancer.

| Supplemental Table 1. 1-year survival among clinical stage II patients (n=296) | | | | | |
| --- | --- | --- | --- | --- | --- |
|  | Baseline | 3 months | 6 months | 9 months | 12 months |
| Lobectomy |  |  |  |  |  |
| Number at risk | 275 | 275 | 269 | 264 | 255 |
| Percent survival | 100% | 100% | 97.82% | 96.00% | 92.73% |
| Standard error | 0 | 0 | 0.0088 | 0.0118 | 0.0157 |
| Sublobar-overall |  |  |  |  |  |
| Number at risk | 21 | 21 | 21 | 21 | 20 |
| Percent survival | 100% | 100% | 100% | 100% | 95.24% |
| Standard error | 0 | 0 | 0 | 0 | 0.0465 |
| Sublobar-wedge |  |  |  |  |  |
| Number at risk | 8 | 8 | 8 | 8 | 7 |
| Percent survival | 100% | 100% | 100% | 100% | 87.5% |
| Standard error | 0 | 0 | 0 | 0 | 0.1169 |
| Sublobar-segment |  |  |  |  |  |
| Number at risk | 13 | 13 | 13 | 13 | 13 |
| Percent survival | 100% | 100% | 100% | 100% | 100% |
| Standard error | 0 | 0 | 0 | 0 | 0 |
