# Supplementary material for: Survival following sublobar resection after neoadjuvant therapy for T1N1-2M0 lung cancer
Source: PLoS One. 2026 Jun 3;21(6):e0349231. doi: 10.1371/journal.pone.0349231 (PMC13232803; doi:10.1371/journal.pone.0349231)
Supplement: S2 Table — Kaplan–Meier curves show 1‑year overall survival for clinical stage III NSCLC patients (n = 1,943). At baseline, numbers at risk included 1,819 for lobectomy and 124 for sublobar resections. One‑year survival was 93.74% for lobectomy compared with 91.08% in the sublobar overall cohort. Among sublobar techniques, wedge resection demonstrated 93.5% 1‑year survival and segmentectomy 91.4%. Standard errors are reported at each interval to quantify uncertainty. (DOCX) [file pone.0349231.s002.docx]

Supplemental Data — Survival Following Sublobar Resection After Neoadjuvant Therapy for T1N1–2M0 Lung Cancer.

| Supplemental Table 2. 1-year survival among clinical stage III patients (n=1943) | | | | | |
| --- | --- | --- | --- | --- | --- |
|  | Baseline | 3 months | 6 months | 9 months | 12 months |
| Lobectomy |  |  |  |  |  |
| Number at risk | 1819 | 1819 | 1789 | 1732 | 1678 |
| Percent survival | 100% | 100% | 98.73% | 96.13% | 93.74% |
| Standard error | 0 | 0 | 0.0026 | 0.0045 | 0.0057 |
| Sublobar-overall |  |  |  |  |  |
| Number at risk | 124 | 124 | 123 | 118 | 112 |
| Percent survival | 100% | 100% | 99.19% | 95.95% | 91.08% |
| Standard error | 0 | 0 | 0.0080 | 0.0177 | 0.0256 |
| Sublobar- wedge |  |  |  |  |  |
| Number at risk | 47 | 47 | 47 | 46 | 43 |
| Percent survival | 100% | 100% | 100% | 100% | 93.5% |
| Standard error | 0 | 0 | 0 | 0 | 0.0364 |
| Sublobar- segment |  |  |  |  |  |
| Number at risk | 70 | 70 | 69 | 66 | 64 |
| Percent survival | 100% | 100% | 98.6% | 94.3% | 91.4% |
| Standard error | 0 | 0 | 0.014 | 0.0277 | 0.0335 |
