# Supplementary material for: Survival following sublobar resection after neoadjuvant therapy for T1N1-2M0 lung cancer
Source: PLoS One. 2026 Jun 3;21(6):e0349231. doi: 10.1371/journal.pone.0349231 (PMC13232803; doi:10.1371/journal.pone.0349231)
Supplement: S3 Table — Kaplan–Meier curves depict 5‑year overall survival for clinical stage II NSCLC patients (n = 296). At baseline, 275 patients were at risk in the lobectomy group and 21 in the sublobar group. Five‑year survival was 56.56% following lobectomy and 40.48% for the sublobar overall group. Subgroup survival estimates at 5 years were 35.0% for wedge resection and 48.9% for anatomic segmentectomy. Standard errors accompany all estimates to indicate statistical precision. (DOCX) [file pone.0349231.s003.docx]

Supplemental Data — Survival Following Sublobar Resection After Neoadjuvant Therapy for T1N1–2M0 Lung Cancer.

| Supplemental Table 3. 5-year survival among clinical stage II patients (n=296) | | | | | | |
| --- | --- | --- | --- | --- | --- | --- |
|  | Baseline | Year 1 | Year 2 | Year 3 | Year 4 | Year 5 |
| Lobectomy |  |  |  |  |  |  |
| Number at risk | 275 | 255 | 206 | 153 | 118 | 86 |
| Percent survival | 100% | 92.73% | 80.41% | 72.58% | 64.71% | 56.56% |
| Standard error | 0 | 0.0157 | 0.0242 | 0.0280 | 0.0311 | 0.0340 |
| Sublobar-overall |  |  |  |  |  |  |
| Number at risk | 21 | 20 | 14 | 10 | 6 | 5 |
| Percent survival | 100% | 95.24% | 70.83% | 70.83% | 48.57% | 40.48% |
| Standard error | 0 | 0.0465 | 0.1005 | 0.1005 | 0.1275 | 0.1294 |
| Sublobar-wedge |  |  |  |  |  |  |
| Number at risk | 8 | 7 | 7 | 5 | 3 | 2 |
| Percent survival | 100% | 87.5% | 87.5% | 87.5% | 52.5% | 35.0% |
| Standard error | 0 | 0.1169 | 0.1169 | 0.1169 | 0.2041 | 0.1973 |
| Sublobar-segment |  |  |  |  |  |  |
| Number at risk | 13 | 13 | 7 | 5 | 3 | 3 |
| Percent survival | 100% | 100% | 59.8% | 59.8% | 48.9% | 48.9% |
| Standard error | 0 | 0 | 0.1401 | 0.1401 | 0.1550 | 0.1550 |
