# Supplementary material for: Survival following sublobar resection after neoadjuvant therapy for T1N1-2M0 lung cancer
Source: PLoS One. 2026 Jun 3;21(6):e0349231. doi: 10.1371/journal.pone.0349231 (PMC13232803; doi:10.1371/journal.pone.0349231)
Supplement: S4 Table — Kaplan–Meier curves illustrate 5‑year overall survival in clinical stage III NSCLC patients (n = 1,943) treated with lobectomy or sublobar resection. At baseline, numbers at risk were 1,819 for lobectomy and 124 for sublobar procedures. Five‑year survival was 59.85% for lobectomy compared with 48.62% for sublobar resection overall. For sublobar subtypes, 5‑year survival was 48.0% after wedge resection and 47.7% after segmentectomy. Standard errors are shown for all survival points. (DOCX) [file pone.0349231.s004.docx]

Supplemental Data — Survival Following Sublobar Resection After Neoadjuvant Therapy for T1N1–2M0 Lung Cancer.

| Supplemental Table 4. 5-year survival among clinical stage III patients (n=1943) | | | | | | |
| --- | --- | --- | --- | --- | --- | --- |
|  | Baseline | Year 1 | Year 2 | Year 3 | Year 4 | Year 5 |
| Lobectomy |  |  |  |  |  |  |
| Number at risk | 1819 | 1678 | 1418 | 1154 | 913 | 719 |
| Percent survival | 100% | 93.74% | 82.67% | 73.42% | 65.18% | 59.85% |
| Standard error | 0 | 0.0057 | 0.0090 | 0.0107 | 0.0118 | 0.0125 |
| Sublobar-overall |  |  |  |  |  |  |
| Number at risk | 124 | 112 | 90 | 63 | 50 | 39 |
| Percent survival | 100% | 91.08% | 73.95% | 61.37% | 52.21% | 48.62% |
| Standard error | 0 | 0.0257 | 0.0396 | 0.0450 | 0.0476 | 0.0486 |
| Sublobar- wedge |  |  |  |  |  |  |
| Number at risk | 47 | 43 | 36 | 24 | 20 | 12 |
| Percent survival | 100% | 93.5% | 78.3% | 64.3% | 58.8% | 48.0% |
| Standard error | 0 | 0.0364 | 0.0608 | 0.0721 | 0.0756 | 0.0837 |
| Sublobar- segment |  |  |  |  |  |  |
| Number at risk | 70 | 64 | 50 | 36 | 27 | 24 |
| Percent survival | 100% | 91.4% | 72.8% | 60.0% | 47.7% | 47.7% |
| Standard error | 0 | 0.0335 | 0.0534 | 0.0602 | 0.0633 | 0.0633 |
